# Nerves Around the Shoulder: What the Radiologist Should Know?

**DOI:** 10.5334/jbr-btr.1382

**Published:** 2017-12-16

**Authors:** Afarine Madani, Viviane Creteur

**Affiliations:** 1 CUB Erasme Hospital, BE

**Keywords:** Peripheral neuropathy, Imaging, shoulder

## Abstract

Peripheral neuropathies of the shoulder are common and could be related to traumatic injury, shoulder surgery, infection or tumour but usually they result from an entrapment syndrome. Imaging plays an important role to detect the underlying causes, to assess the precise topography and the severity of nerve damage. The key points concerning the imaging of nerve entrapment syndrome are the knowledge of the particular topography of the injured nerve, and the morphology as well signal modifications of the corresponding muscles. Magnetic Resonance Imaging best shows these findings, although Ultrasounds and Computed Tomography sometimes allow the diagnosis of neuropathy.

## Introduction

Peripheral neuropathies of the shoulder are common and represent an important cause of morbidity and disability in patients. They could be related to traumatic injury, shoulder surgery, infection or tumour, but usually they result from an entrapment syndrome, a condition in which the nerve is stretched into an incompressible space [1].

Although all the nerves of the shoulder could be affected, we will focus our topic on the major nerves that could be involved in this area, principally suprascapular nerve, followed by the axillary, musculocutaneous, spinal accessory and long thoracic nerves. The sensory and motor innervations of these nerves as well as the etiology and usual site of entrapment are summarized in Table 1.

**Table 1:** Summary of shoulder neuropathy.

Nerve involved	Sensory innervation	Motor innervation	Usual site of entrapment	Etiology

Suprascapular (C4)–C5–C6 roots	Acromioclavicular joint Glenohumeral joint Subacromial bursa	Supraspinatus and Infraspinatus	Suprascapular notch	Trauma, Microtrauma, Surgery, Extrinsic compression (cyst, tumour, varicose, etc.), Rotator cuff tears
		Isolated Infraspinatus	Distal from suprascapular notch: spinoglenoid notch	
Axillar C5–C6 roots	Glenohumeral joint Superior lateral brachial cutaneous nerve	Teres Minor and Deltoid	Quadrilateral space	Trauma (shoulder dislocation, humeral surgical neck fracture), Microtrauma, Surgery, Extrinsic compression (hematoma, posteroinferior labral cyst, bony callus, tumour, etc.)
		Isolated Deltoid (anterior and middle heads)	Anterior branch	
		Isolated Teres Minor		
		Isolated Deltoid (posterior head)	Posterior branch	
Musculocutaneous (C4)–C5–C6 (C7) roots	Lateral antebrachial cutaneous nerve	Biceps Brachii- Coracobrachialis-Brachialis	Proximal coracobrachialis	Trauma, Microtrauma, Anterior shoulder surgery (Latarjet)
Long thoracic nerve (C4) C5–C6–C7 roots	/	Serratus Anterior (Winging scapula)	Scalenus medius muscle Second rib	Trauma, Microtrauma, Surgery (mastectomy, scalenectomy) Extrinsic compression (hematoma, tumour, etc)
Spinal accessory nerve (Cranial nerve XI–C1–C5)	/	Trapezus Sternocleidomastoid (Droopy Shoulder)	Posterior triangle of the neck	Trauma Microtrauma Surgery (radical neck tumour dissection, etc) Extrinsic compression (hematoma, tumour, etc)
		Trapezus	Posterior to sternocleidomastoid muscle	

**Figure 1:**
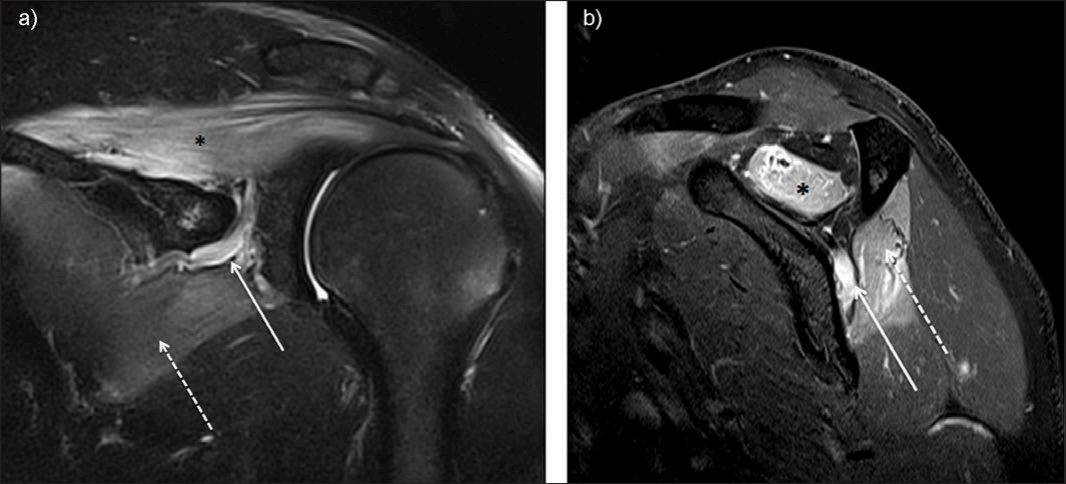
Suprascapular neuropathy at the scapular notch in a young judoka athlete showing typical edema denervation pattern of acute neuropathy on MRI. High signal intensity fluid is observed in fluid-sensitive sequences of both supraspinatus (star) and infraspinatus (dashed arrow). Notice the dilatation of suprascapular veins satellite (arrow). (**1a** = FIGURE 1 uploaded online manuscript) – Coronal (**1b** = FIGURE 2 uploaded online manuscript) – sagittal Short Tau Inversion recovery MRI (STIR) images.

Imaging plays an important role to detect the underlying causes of nerve damage. Conventional X-Ray is still useful in shoulder neuropathy diagnosis by assessing glenohumeral and acromioclavicular joints, by excluding cervical ribs, by evaluating the cervical spine with underlying discopathies, by excluding bone fracture or joint dislocation in case of trauma and by assessing bone surgery and orthopedic materials. Computed tomography (CT) could add more information than X-ray by allowing morphologic evaluation of suprascapular and spinoglenoid notches, assessing the muscular trophicity and fatty degeneration, and showing extrinsic compression of the injured nerve by labral, mucoid cysts or tumours. Ultrasonography (US) is a useful, non-invasive modality that allows bilateral with dynamic comparison of the periscapular soft tissue. US does sometimes allow morphologic assessment of the nerves, essentially on the basis of focal thickening, and can also guide diagnosis on the basis of atrophy and fatty degeneration, characterized by a loss of muscle volume and muscle hyperechogenicity. The key points concerning the imaging of nerve entrapment syndrome are the morphology and the signal changes of the innervated muscles, best demonstrated by Magnetic Resonance Imaging (MRI). The earliest MRI sign of an impairment of the relationship between motor neurons and muscle fibers is the *edema* signal of the denervated muscle. This finding is related to an increased extracellular free water and muscle blood volume. In acute and subacute stages, high signal intensity fluid is found in T2-weighted with fat suppression sequences associated with normal signal in T1-weighted sequences (Figure 1). In chronic denervation cases, atrophy and fatty degeneration are the main signs.

## Suprascapular Nerve

### Normal anatomy

**Figure 2:**
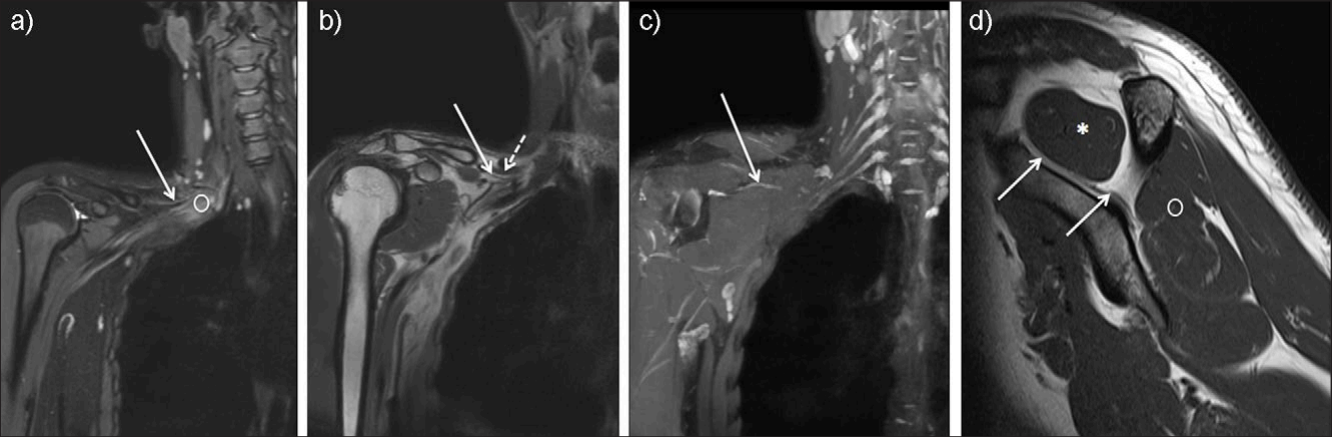
Coronal MR Neurography sections showing the right suprascapular nerve (solid arrow) arising from the upper trunk of the plexus brachial (circle) (**2a** = FIGURE 3 uploaded online manuscript) then, crossing the posterior cervical triangle in the supraclavicular fossa, deep to the omohyoid muscle (dashed arrow) (**2b** = FIGURE 4 uploaded online manuscript), before traveling through the suprascapular notch (**2c** = FIGURE 5 uploaded online manuscript). Sagittal MRI T1-weighted sequence shows the nerve inside the supraspinatus fossa where it provides motor innervation to the supraspinatus muscle (star) followed by infraspinatus muscle (circle) (**2d** = FIGURE 6 uploaded online manuscript).

The suprascapular nerve is a mixed nerve providing motor innervation to the supraspinatus and infraspinatus muscles and sensory innervation of the coracohumeral ligament, the coracoclavicular ligament, the subacromial bursa, the acromioclavicular joint and upper and posterior glenohumeral joint. The nerve arises from the upper trunk of the plexus brachial and is formed by the ventral rami of C5 and C6 roots and occasionally from the C4 root. Then, it crosses the posterior cervical triangle in the supraclavicular fossa, deep to the omohyoid muscle (Figure 2) [2,3]. In a recent study carried out in 30 healthy volunteers and five cadavers by Laumonerie, et al., the diameter of the suprascapular nerve at its origin averaged 1.33 mm (range, 1–1.9 mm) and its depth averaged 5.12 mm (range, 2.7 mm–10.6 mm) using ultrasound technique [4]. In another study, carried out in 15 healthy volunteers, by Gruber, et al., the cross-sectional area of the suprascapular nerve averaged 2.9 ± 0.9 mm² at its origin and 2.79 ± 0.83 mm² at the level of the omohyoid muscle [5]. Distally, the nerve travels through the suprascapular notch under the superior transverse ligament, inconstantly accompanied by suprascapular artery and vein. Then, it enters the supraspinatus fossa where it provides motor innervation to the supraspinatus muscle. The sensitive components of the nerve, providing about 70% of shoulder articular sensitivity, emerge from the suprascapular nerve just before and after passing below superior transverse ligament [6]. The nerve passes then through the spinoglenoid notch, under the spinoglenoid ligament, where it supplies motor branches to the infraspinatus muscle.

### Common and uncommon pathological findings

The clinical presentation of suprascapular neuropathy may be various. The pain is usually chronic and dull, in the superior and posterior shoulder often radiation to the neck or lateral arm. Weakness, loss of function and atrophy of the shoulder are other clinical manifestations.

The causes of the nerve entrapment are multiple. The suprascapular notch is the most frequent point for the suprascapular nerve to be entrapped by compression or traction. In addition, the anatomic configuration of the suprascapular notch may represent a predisposing factor to the development of entrapment. Several morphologic variations in size and shape of the suprascapular notch have been reported. Rengachary et al., described six types of suprascapular notch and their incidence, based on the shape of the notch [7]. Natsis et al., proposed a simplified classification, on the basis of vertical and the transverse diameters of the suprascapular notch [8]. Other predisposing factors are uncommon narrow or closed notches and the ossification of the transverse suprascapular ligament, the latter being related to aging [9,10]. The role of the coracoscapular ligament as a possible factor for nerve entrapment remains unclear [11,12].

**Figure 3:**
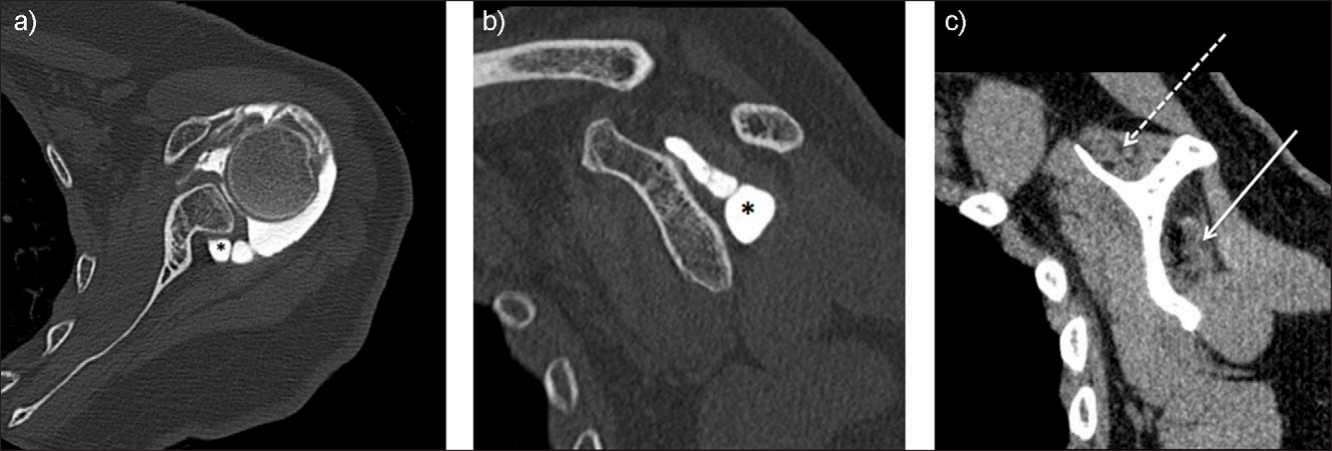
Neuropathy of suprascapular nerve by voluminous labral cyst (star) sited in spinoglenoid notch extending to supraspinatus fossa inducing a severe atrophy and fatty degeneration of infraspinatus (solid arrow) whereas the supraspinatus muscle is less involved (dashed arrow). Axial (**3a** = FIGURE 7 uploaded online manuscript) – sagittal (**3b** = FIGURE 8 uploaded online manuscript – **3c** = FIGURE 9 uploaded online manuscript) CT Arthrography.

Extrinsic compression by labral or mucoïd cyst (Figure 3), tumour or varicose enlargement of the suprascapular veins (Figure 1), is another common cause of nerve entrapment. Fractures of the suprascapular notch, or posterior fracture dislocation of the humeral head, have been associated with lesions of suprascapular nerve [13].

Microtraumatisms by repetitive movements, especially with overhead activities, for example in volleyball and baseball athletes, expose the nerve to tension especially with a predisposing anatomy. As the shoulder is in extreme external rotation and abduction, the muscles of supraspinatus and infraspinatus impinge upon the scapular spine compressing the motor branch of the infraspinatus, resulting to an isolated infraspinatus muscle weakness [14].

At the level of the spinoglenoid notch, the nerve entrapment may be due to an increased tension in adduction and internal rotation with a hypertrophied inferior transverse scapular ligament [1].

A possible association between the suprascapular nerve entrapment and extensive rotator cuff tears has been reported. The mechanism of this entrapment is based on cadaveric study that showed that the medial retraction of supraspinatus tendon stretches the nerve by changing the angle between the nerve and its first motor branch [15]. This relationship is supported by clinical studies showing the recovery of nerve entrapment after cuff tear repair [16,17].

Imaging, especially MRI, plays an important role to detect the underlying causes of nerve damage (extrinsic mass, rotator cuff tear, etc.), the precise topography of injury (involvement of both supraspinatus and infraspinatus muscles if the nerve is injured at suprascapular notch and isolated denervation sign of infraspinatus muscle if the nerve is injured distally to suprascapular notch) MRI is also useful to determine the severity of the nerve injury (edema and/or atrophy) and to diagnose other cause of neuropathy such as cervical radiculopathy or Parsonage Turner syndrome. This syndrome is a rare idiopathic disorder, often attributed to viral infections or immunological reaction after vaccination, characterized by a sudden onset of pain of the shoulder girdle affecting one or several nerves of brachial plexus [18]. There is no consensus regarding the most peripheral nerve frequently affected, but the suprascapular nerve is involved frequently [19]. Bilateral or multiple involvements of muscles without relevant trauma or sport activities or heterogeneous patterns of muscle signal on MRI are important clues for ruling out Parsonage Turner syndrome. Recently, Sneag et al., reported the MRI *bullseye sign* as an indicator of peripheral nerve constriction in Parsonage Turner syndrome [20].

## Axillary Nerve

### Normal anatomy

The axillary nerve is a mixed nerve, providing motor innervation of the deltoid and teres minor muscles, supplying collateral branches to the subscapularis and coracobrachialis muscles. This nerve provides also sensory innervation of the shoulder joint capsule (shared with suprascapular nerve), the inferior glenohumeral ligament and the posterolateral skin of the shoulder and the arm. The nerve is the terminal branch of posterior cord of the plexus brachial and is formed by the ventral rami of C5 and C6 roots, lying superior to the radial nerve and behind the axillary artery and vein [21,22]. Five anatomic segments have been proposed by Duparc et al., in order to determine the complex course of this nerve. The nerve runs obliquely in front of the subscapularis muscle, crossing the inferior border of this muscle (segment 1), courses to the anterolateral border of the tendon of long head of triceps brachii (segment 2), then to the posteromedial part of surgical neck of the humerus (segment 3) in the quadrilateral space. After crossing the humerus, the nerve enters in the deltoid muscle (segment 4) and then courses inside the deltoid muscle (segment 5) [23,24].

The quadrilateral space, also called quadrangular space or lateral axillary hiatus, is bounded by the teres minor muscle superiorly, the upper border of the teres major muscle inferiorly, the long head of the triceps brachii medially, and the surgical neck of humerus laterally [25]. In most cases, the axillary nerve divides into anterior and posterior branches inside the quadrilateral space. The anterior branch ascends around the surgical neck of humerus and provides innervation of anterior and middle part of the deltoid by numerous branches; the posterior branch innervates the teres minor via a single branch, the posterior part of the deltoid muscle and continues as the superior lateral brachial cutaneous nerve [22,23].

The differences in innervation between the anterior and posterior branches of the axillary nerve explain the occurrence of an isolated palsy of the deltoid or the teres minor muscles with or without sensory loss in the axillary nerve distribution [22].

### Common and uncommon pathological findings

**Figure 4:**
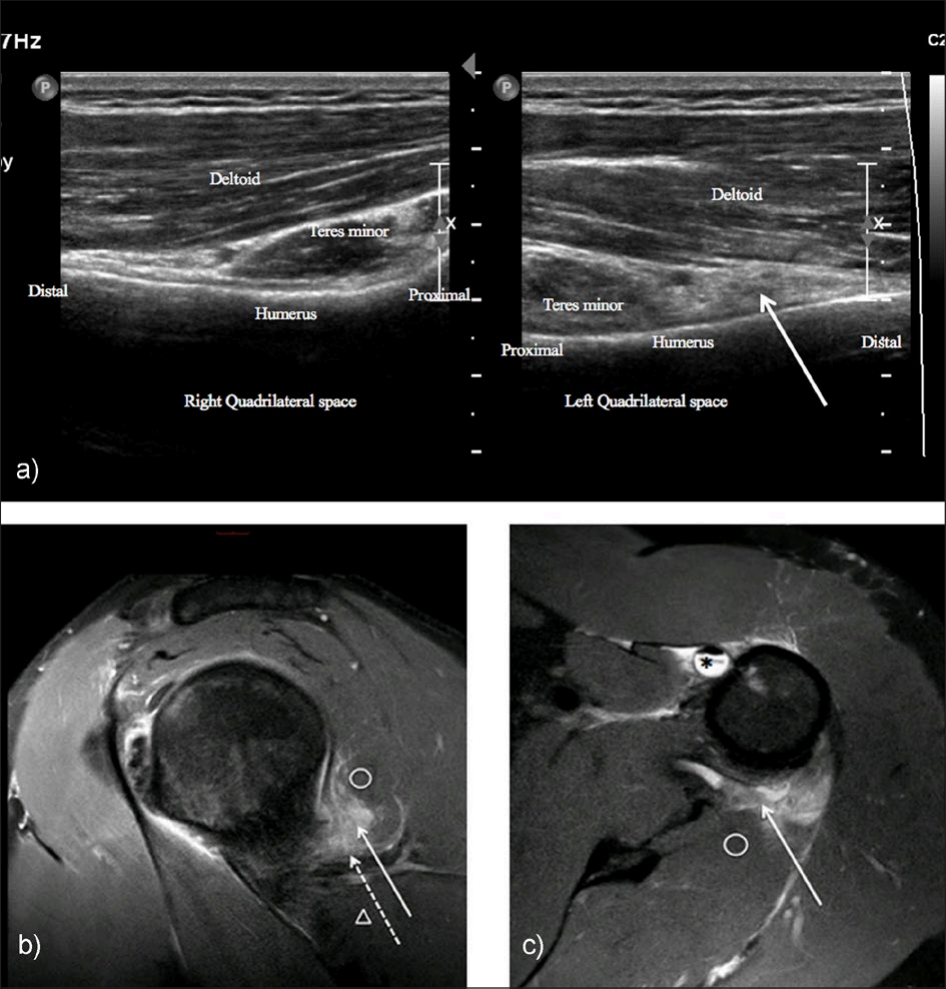
Neuropathy of axillary nerve after skiboard fall leading to injury of the shoulder capsule. Ultrasound showed infiltration of the axillary nerve (dashed arrow) in quadrilateral space (solid arrow) compared to normal side (**4a** = FIGURE 10 uploaded online manuscript), confirming by sagittal (**4b** = FIGURE 11 uploaded online manuscript), axial (**4c** = FIGURE 12 uploaded online manuscript) Proton Density with Fat Saturation MRI images. Note the concomitant Biceps brachii tendon injury (star). Teres minor muscle (circle) – Teres major muscle (arrowhead).

The axillary nerve injury is one of the most commonly injured nerves during surgical procedures of the shoulder making up to 10% of all brachial plexus injuries. The anterior branch of the nerve, ascending around the surgical neck of humerus is at risk during deltoid splitting or humeral nailing or prosthetic procedures [26]. It may also be injured during acute trauma to the shoulder, especially in surgical neck fracture of humerus, in shoulder dislocation or in direct anterolateral blow to the deltoid muscle (Figure 4). Occurring in 9–18% of patients with traumatic shoulder dislocation, the *unhappy triad* is the condition in which multiple peripheral nerves injury occurs in combination with rotator cuff tear. In this triad, the axillary nerve is the most commonly affected (47% only axillary; 45% axillary and musculocutaneous; 36% axillary and suprascapular; 6% axillary, suprascapular and long thoracic nerves) [27].

Extrinsic compression by hematoma, posteroinferior labral cyst, bone callus, tumour, and accessory subscapularis muscle are other common etiologies of axillary nerve injury [1,28].

Chronic compression of the axillary nerve, known as quadrilateral space syndrome is a rare and misdiagnosed neurovascular syndrome. This syndrome is defined as compression or mechanical injury of the axillary nerve or posterior circumflex artery as they pass through the quadrilateral space. The clinical manifestation is various including nondermatomal neuropathic pain, numbness and weakness in the shoulder or vascular manifestations such as thrombosis, digital or hand ischemia. The electromyography is often normal. This syndrome has been reported in overhead or throwing athlete including volleyball, baseball, swimming but also in yoga or window cleaning that involve abduction and external rotation [25]. Imaging can play an important role in detecting the underlying causes of nerve damage by showing extrinsic mass, or deltoid or teres minor muscles denervation sign. It should also be noticed that about 50% of Parsonage Turner cases affect the axillary nerve and thus this diagnosis must be kept in mind.

## Musculocutaneous Nerve

### Normal anatomy

The musculocutaneous nerve is a mixed nerve, providing motor innervation to the coracobrachialis, biceps brachii, and brachialis muscles and sensory innervation of lateral forearm. The nerve arises from the lateral cord of the plexus brachial and is formed by the ventral rami of C5 and C6 roots and occasionally from the C4 or C7 roots. There are numerous anatomical variations. Located lateral to the median nerve and axillary artery, the nerve runs frequently downward and passes through the coracobrachialis muscle and descends obliquely between the biceps brachii and the brachialis muscles, which it innervates. Then, the nerve emerges along the lateral margin of biceps aponeurosis and continues in the forearm as the lateral antebrachial cutaneous nerve [29].

### Common and uncommon pathological findings

Weakness of biceps brachii muscle and, sometimes, sensory deficit in the forearm are the main symptoms of musculocutaneous neuropathy. Isolated musculocutaneous nerve injuries are rare and the reported causes are penetrating traumas, anesthetic blocks, anterior shoulder surgery especially in coracoid abutment (Latarjet procedure), and sport related entrapment such as windsurfing, rowing or weightlifting athletes. Proximal nerve injury can occur when the nerve pierces the coracobrachialis muscle during violent extension of the arm in throwing athletes or by entrapment between the biceps brachii and the brachialis muscles, in forced abduction and external rotation [29,30,31]. Imaging, especially ultrasound, can be useful to assess the nerve after surgical procedure, and to exclude cervical radiculopathy.

## Long Thoracic Nerve

### Normal anatomy

The long thoracic nerve (Charles Bell nerve) is a pure motor nerve arising directly from the ventral rami of C5, C6, and C7 roots and occasionally from the C4 root. The roots from C5 and C6 runs downward and pierce through the scalenus medius, while the root from C7 runs over this muscle. In the supraclavicular region, the upper division of the long thoracic nerve runs parallel and posterior to the brachial plexus close to the supra- scapular nerve. In the axilla, the upper and lower portion merge and extends along the side of the thorax to the lower border of the serratus anterior, supplying filaments to each digitation of this muscle [32].

### Common and uncommon pathological findings

Traumatic injuries to the nerve after motor vehicle accidents, or after falls have been reported [30]. The nerve injury may occur at two critical points: at the level of scalenus medius muscle or at the level of the second rib. Sports related nerve injuries by excessive traction between these two-fixed points have also been reported in weightlifting, volleyball, and javelin throwing and bodybuilding athletes [34]. Iatrogenic injury to the nerve is not uncommon especially in first rib resection, mastectomy with axillary dissection, scalenectomy, and infraclavicular plexus anesthesia [33] could induce long thoracic nerve damage leading to serratus anterior impairment and winged scapula. Scapular winging is a rare but painful and debilitating condition, characterized by a failure of dynamic stabilizing structures that keep the scapula anchored to the chest wall, leading to a prominence of the scapula [34,35]. The major scapulothoracic stabilizing structures include serratus anterior muscle, trapezius and rhomboids muscles. Serratus anterior palsy is the most diagnosed form of scapular winging leading to downward tilting of the acromion and the inability to release the acromion from the greater tuberosity during shoulder elevation. The incidence of scapular winging is unclear, but often missed because it can mimic other shoulder disorders such as rotator cuff disorders, glenohumeral instability, cervical spine disorders, or acromioclavicular disorders.

Imaging shows denervation signs (edema and atrophy) of the serratus anterior and may help to eliminate other causes of scapular winging. In case of multiple muscle denervation sites, imaging could suggest a Parsonage Turner syndrome.

## Spinal Accessory Nerve

### Normal anatomy

The spinal accessory nerve (cranial nerve XI) provides motor innervation of the trapezius and sternocleidomastoid muscles. It is a motor nerve arising from both the medulla and the spinal cord. The cranial fibers innerve the pharyngeal and laryngeal muscles and the spinal fibers arise from the anterior horn of the upper five (or six) cervical vertebra. The spinal fibers enter the posterior cranial fossa, merge with the cranial fibers and then exit the skull via the jugular foramen, along with the vagus and glossopharyngeal nerves. The nerve passes deep into the posterior belly of the digastric muscle to supply the sternocleidomastoid muscle. Then, it passes through this muscle and runs obliquely across the posterior cervical to end in the deep surface of trapezius muscle via several terminal branches to supply upper, middle and lower trapezius muscle [36,37].

### Common and uncommon pathological findings

The nerve is vulnerable in the posterior triangle of the neck, during radical neck surgery, tumour dissection and cervical lymph node biopsy. Traumatic injury by direct impact or deep tissue massage and water-skiing injuries has been reported but occurs rarely. These injuries lead to a *droopy shoulder* characterized by the inability to raise the affected arm above the level of the shoulder.

**Figure 5:**
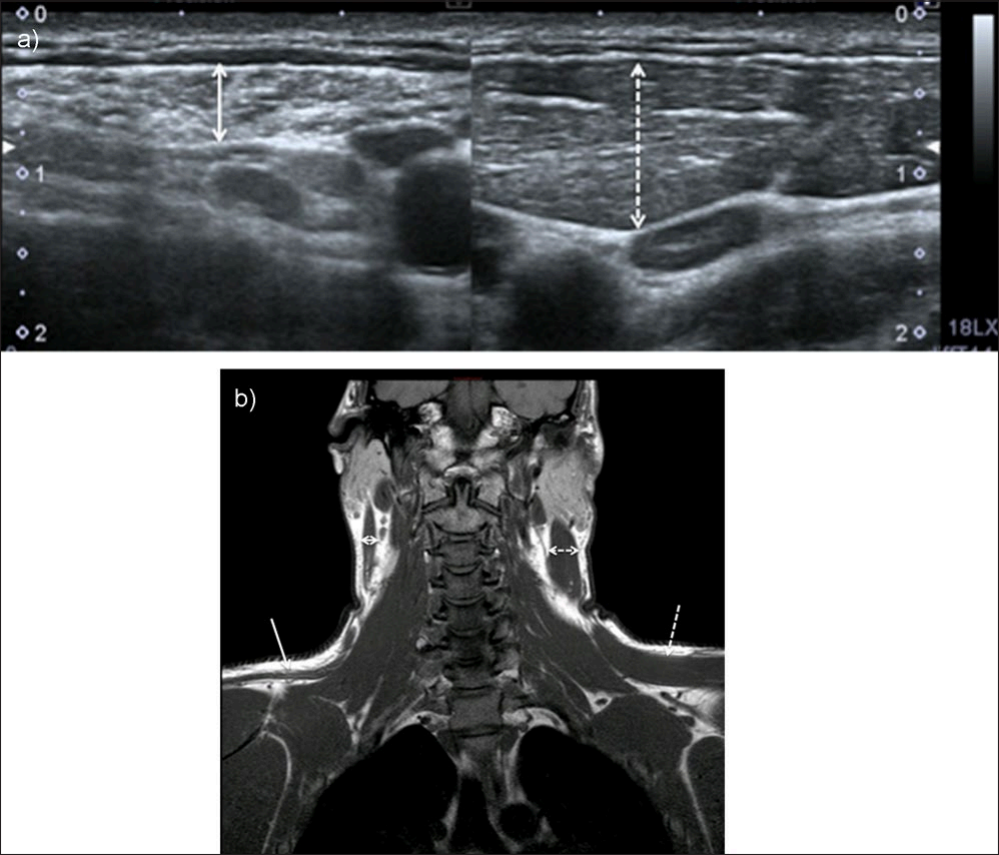
Chronic neuropathy of spinal accessory nerve. Ultrasound showed atrophy of sternocleidomastoid muscle (double solid arrow) compared to normal side (doubled dashed arrow) (**5a** = FIGURE 13 uploaded online manuscript), confirmed by coronal T1-weighted MRI showing both atrophy of trapezus (solid arrow) and sternocleidomastoid muscles compared to normal side (dashed arrow) (**5b** = FIGURE 14 uploaded online manuscript).

Imaging of this nerve is quite challenging but MRI shows denervation of the trapezius and sternocleidomastoid muscles (Figure 5). In patients who have had posterior triangle neck surgery, scarring may be seen around the nerve [37].

## Summary

Diagnosis of shoulder neuropathy is difficult and challenging because of overlapping symptoms with various origins. Imaging plays an important role to detect the underlying causes to assess the precise topography and the severity of nerve damage. The key points concerning the imaging of nerve entrapment syndrome are the knowledge of the precise topography of the injured nerve and the morphological and signal change of the innervated muscle, best shown by MRI, although US and CT may allow the diagnosis of neuropathy.
